# Influence of iris color on corneal densitometry

**DOI:** 10.1016/j.optom.2025.100595

**Published:** 2026-01-22

**Authors:** Ana R. Arizcuren, Alejandra Consejo

**Affiliations:** Aragon Institute for Engineering Research (I3A), University of Zaragoza, Zaragoza, Spain

**Keywords:** Corneal densitometry, Corneal transparency, Iris color, Scheimpflug imaging, Image processing, Backscattering

## Abstract

**Purpose:**

To determine whether iris pigmentation introduces a measurable bias in corneal densitometry (CD) values obtained via Scheimpflug imaging, and to develop an objective metric for iris color quantification.

**Methods:**

This observational study included 91 eyes from 47 healthy adults. CD was assessed as mean pixel intensity (MPI) from 25 Scheimpflug images per eye. Brightness artefacts from the iris were quantified using automated image processing. Iris color was objectively characterized from slit-lamp photographs using an objective single-value metric (*IrisColor*) derived from normalized CIELAB components. Associations among CD, iris brightness, and iris pigmentation were evaluated using Pearson correlation and linear mixed-effects models (LMMs).

**Results:**

CD correlated positively with iris brightness artefacts (*r* = 0.47, *β* = 1.49, *p* < 0.001), which in turn showed a strong negative correlation with *IrisColor* (*r* = –0.83, *β* = –1.11, *p* < 0.001). Lighter-colored irises (lower *IrisColor* values) exhibited statistically significantly higher CD values equivalent to a 6.6% relative overestimation. Groupwise comparisons confirmed that iris pigmentation significantly influences both CD and overall image brightness.

**Conclusion:**

Iris pigmentation induces a measurable bias in Scheimpflug-based CD estimates, primarily through increased brightness artefacts in light-colored eyes. The proposed *IrisColor* metric enables objective, continuous classification of iris color and could support future corrections for pigmentation-induced bias in CD-based diagnostics.

## Introduction

Corneal transparency is a key determinant of optical quality and visual performance. In recent years, corneal densitometry (CD) has emerged as a robust imaging-based tool to evaluate transparency by quantifying light backscatter across the corneal tissue. Applications of CD include the monitoring of corneal diseases,^1^^,^^2^ the evaluation of surgical outcomes,^3^^,^^4^ and the early detection of disorders such as keratoconus.^5^^,^^6^

**Fig. 1:**
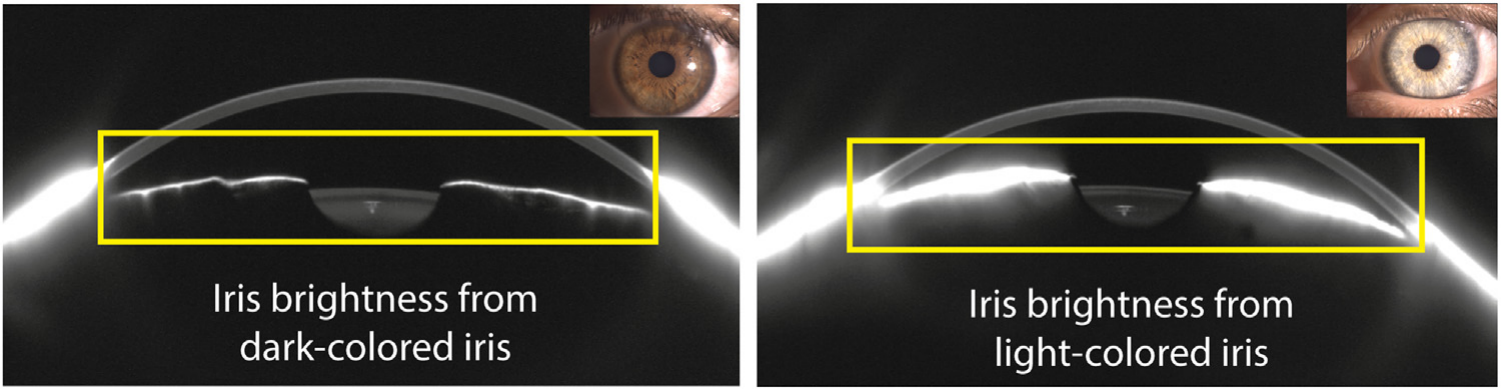
Example of brightness artifacts from iris reflections (yellow rectangles) in a dark-colored iris (left) and a light-colored iris (right).

However, as CD becomes more widely adopted, its sensitivity to external influences has drawn increasing scrutiny. Various factors unrelated to intrinsic tissue properties can distort CD values, potentially confounding clinical interpretations. These include patient age,^7^^,^^8^ ocular biometry (such as anterior chamber depth and pupil size),^9^^,^^10^ epithelial optical properties,^11^ and even the alignment or tilt of the eye during image acquisition.^12^ Additionally, recent work from our group has highlighted the impact of brightness artefacts, i.e., unwanted reflections from surrounding ocular structures, as a significant source of variability in CD estimates.^10^ These artefacts are particularly prominent in Scheimpflug-based tomography due to the wide field of view and strong peripheral reflectance, especially from the sclera and iris.^10^ Additionally, empirically we have observed during imaging that irises with lighter pigmentation tend to produce stronger reflections in Scheimpflug images, whereas darker irises exhibit markedly lower reflectivity. This visual trend, illustrated in Fig. 1, suggests that iris color could introduce systematic differences in the apparent brightness of the anterior segment, potentially biasing CD measurements. Yet, despite these visual trends, the relationship between iris pigmentation and CD measurements has not been formally quantified.

To evaluate whether iris color contributes to variability in CD estimates, an objective and reproducible metric of iris pigmentation is required. Iris color has traditionally been classified subjectively,^13^ often through visual comparison with predefined categories such as “blue,” “hazel-green,” or “brown”,^14^ a process that introduces observer-dependent variability and is further complicated by cultural ambiguity in color terminology. Some studies have attempted to objectively quantify iris pigmentation using RGB values extracted from digital images.^15^ While RGB is the native color space of most imaging devices, its components are highly sensitive to lighting conditions, sensor characteristics, and white balance settings, which can limit consistency and reproducibility. To overcome these limitations, perceptually uniform color spaces such as CIELAB have been proposed.^15^, ^16^, ^17^ CIELAB separates luminance from chromaticity and enables standardized color comparison across different imaging conditions.

In this work, we introduce a straightforward, single-value metric based on the CIELAB color space to classify iris color in a continuous and objective manner. The aim is to assess whether iris pigmentation introduces measurable bias in CD estimates obtained via Scheimpflug imaging.

## Methodology

### Data collection

This study was approved by the Ethics Committee for Clinical Research of Aragon (PI25/184) and adhered to the tenets of the Declaration of Helsinki. All subjects provided written informed consent to participate after the nature and possible consequences of the study had been explained. The current study included 47 Caucasian healthy participants (51% men and 49% women), aged between 21 and 41 years, resulting in measurements for 91 eyes. All participants showed normal visual function, as confirmed by best-corrected visual acuity assessment, and had intraocular pressure within the normal range (15.6 ± 3.0 mmHg), measured with the Corvis ST (https://www.oculus.de/). Eyes included in the study had no history of ocular surgery or corneal pathology, showed no signs of senile arcus, had not worn contact lenses for at least 48 h before the examination, and none of the participants were users of semi-rigid lenses.

All measurements were conducted in a controlled laboratory setting at University of Zaragoza, under identical dark lighting conditions to ensure measurement consistency across participants. Scheimpflug imaging was performed using a Pentacam HR tomographer (https://www.oculus.de/) and served to assess both corneal densitometry, calculated as mean pixel intensity (MPI), and the quantification of spurious brightness artefacts across the image. Additionally, standardized slit-lamp photographs were acquired using a SL650+ biomicroscope (https://www.essilor-instruments.com/) for the objective characterization of iris color. All images were taken at a fixed optical magnification of × 10, with a constant acquisition angle of 55°, under diffuse illumination provided by an integrated diffuser.

### Scheimpflug image processing

**Fig. 2:**
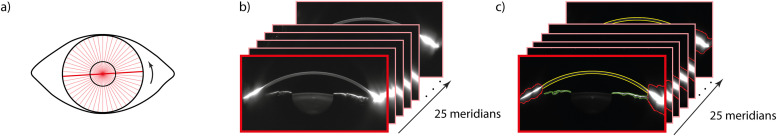
Schematic representation of the Scheimpflug image acquisition and processing. a) The Pentacam HR captures Scheimpflug images along 25 meridians of the anterior segment. b) Example of raw Scheimpflug images. c) Automated segmentation of ocular structures including the cornea (yellow), iris (green), and lateral brightness artifacts (red).

Each measurement with the Pentacam HR tomographer consisted of 25 Scheimpflug images acquired at different meridians (Fig. 2 a-b). Raw Scheimpflug images (i.e., without gamma correction and contrast enhancement applied by default by the Pentacam HR software) were used for analysis. Each image had a fixed resolution of 1200 × 620 pixels. Each image was then segmented and processed to allow for the objective quantification of brightness artefacts (Fig. 2 c). The detailed Scheimpflug image processing is fully described in our previous publication.^10^ Briefly, Scheimpflug images were automatically segmented using standard image processing techniques (median filtering and Canny edge detection) to delineate three primary regions of interest: the cornea, iris, and corneoscleral lateral brightness areas (highlighted in yellow, green and red, respectively, in Fig. 2 c). CD was calculated as the MPI of the segmented cornea in each image (yellow contour in Fig. 2 c), and the final MPI value for each cornea was obtained by averaging the results across all 25 images. From this point forward, the terms MPI and CD will be used interchangeably. As described in previous work,^10^ to quantify brightness artefacts objectively, an adaptive intensity threshold was established individually for each image, defined as the corneal MPI plus three times its standard deviation. The percentage of brightness artefacts was then calculated as the proportion of pixels within each segmented region (iris and lateral brightness) exceeding this threshold, relative to the total pixel count of each respective region. For each eye, the final MPI and brightness artefacts values were obtained by averaging the measurements across all 25 images.

### Metric for iris color objective evaluation (IrisColor)

**Fig. 3:**
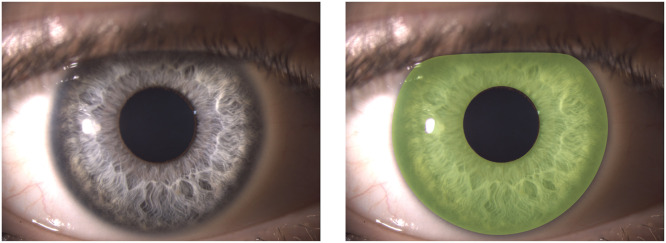
Example of iris segmentation. Original slit-lamp image (left) and the corresponding segmented region (right), shown as a green transparent overlay. Segmentation was performed using the graph cut algorithm implemented in MATLAB's Image Segmenter tool.

To quantify iris color objectively, each slit-lamp image underwent interactive segmentation using the Image Segmenter tool in MATLAB (mathworks.com), applying the graph cut algorithm**.** The region of interest (ROI) was carefully selected to include only the iris, explicitly excluding the pupil, specular highlights, and any non-iris areas (Fig. 3). This step ensured that only the regions corresponding to the iris were analyzed, excluding adjacent structures and artefacts.

Subsequently, each slit-lamp image was converted from RGB to the CIELAB color space. This space separates visual information into three components: L* (lightness), a* (green to red chromaticity), and b* (blue to yellow chromaticity). To summarize iris color as a single scalar value, we applied a multi-step procedure. First, it was necessary to individually normalize each component across the entire data set due to substantial differences in their numerical ranges. Using unnormalized values would have skewed the final metric toward the L* component. Normalization was performed using the median and interquartile range (IQR) of each component to reduce the influence of outliers and better reflect the central tendency of the data. Hence, each component (L*, a*, b*) was normalized using the following:

$$X_{n}=\frac{X-median\left(X\right)}{IQR\left(X\right)}$$

where $X\;\in \;\{L^{*},\;a^{*},\;b^{*}\}$. This produced the normalized variables *L_n_, a_n_*, and *b_n_*. This ensured that all three components contributed comparably and that the process was robust to outliers. During preliminary analyses, it was observed that the L* component exhibited an inverse relationship with the CD, compared to the a* and b* components. While a* and b* decreased with lighter iris pigmentation, L* increased, introducing a directional inconsistency when combining the three channels into a single metric. To resolve this and preserve directional coherence, the normalized L* value was multiplied by (–1) before computing the composite index. This adjustment ensured that all three components contributed in the same direction, this resulted in a single scalar value (*IrisColor*) that objectively characterizes iris color:

$$IrisColor=-L_{n}+a_{n}+b_{n}$$

This value was used for subsequent statistical analysis and correlation with CD. Negative *IrisColor* values correspond to lighter-colored eyes, values near zero indicate intermediate eye colors, and positive values reflect darker irises.

### Statistical analysis

Statistical analysis was performed using MATLAB Statistics and Machine Learning Toolbox. To explore the relationships between the main variables, i.e., corneal densitometry (CD), brightness artefacts from the iris (Iris Spurious Brightness), and iris pigmentation (*IrisColor*) Pearson’s correlation coefficient (*r*) was used to assess the strength and direction of linear associations. Given the continuous nature of these variables, Pearson’s *r* was used to assess the strength and direction of linear associations. However, as some participants contributed both eyes the assumption of independence required for Pearson’s *r* may not be fully met. To account for the potential correlation between fellow eyes, linear mixed-effects models (LMMs) were also fitted, including subject identity as a random intercept, thereby correcting for intra-subject variability while assessing associations between variables. In the LMM framework, the fixed-effect coefficient (*β*) represents the expected change in the dependent variable (e.g., CD) for each unit increase in the predictor (e.g., *IrisColor*), after adjusting for between-subject differences.

In addition, group comparisons were conducted to evaluate differences in CD and brightness artefacts between eyes with light- and dark-colored irises. Group assignment was based on an objective classification using *IrisColor* metric, with negative values corresponding to light-colored irises and positive values to dark-colored irises. Between-group differences were assessed using LMMs with subject as a random effect to correct for potential non-independence. Statistical significance was set at 0.05.

## Results

**Fig. 4:**
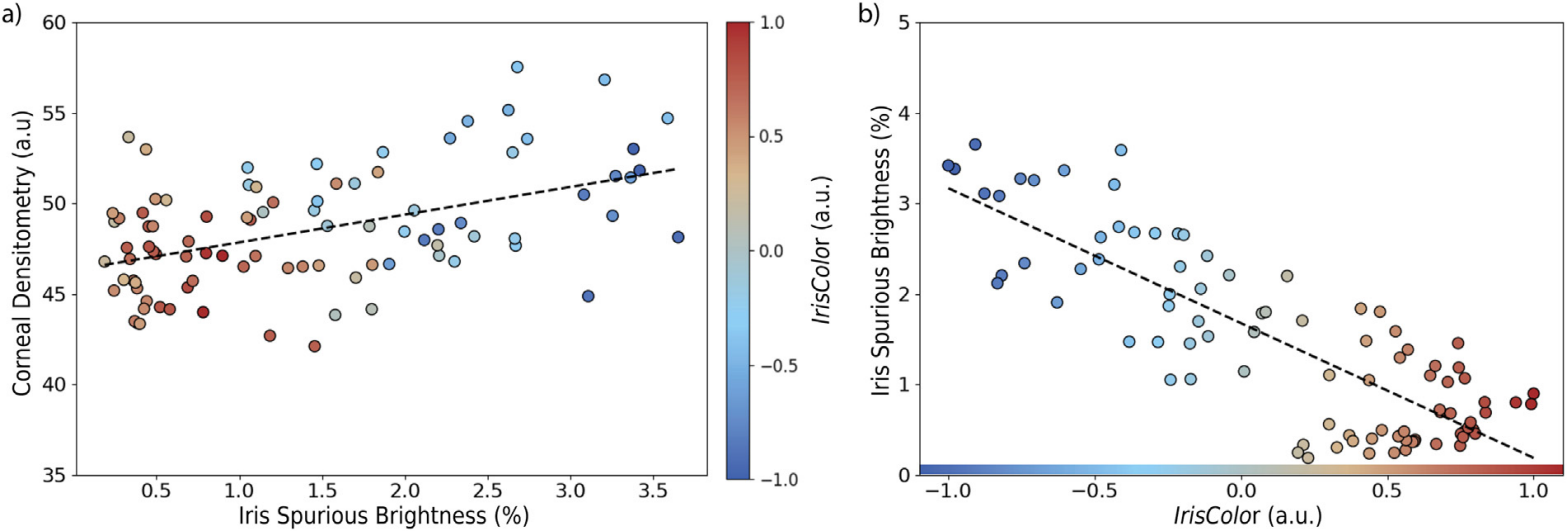
(a) Correlation between corneal densitometry, computed as corneal mean pixel intensity (MPI), and the percentage of spurious brightness originating from the iris (r = 0.47, β = 1.49, p < 0.001). Data points are color-coded according to the continuous IrisColor metric, with negative values representing lighter irises and positive values representing darker irises; (b) Correlation between iris spurious brightness and IrisColor (r = –0.83, β = –1.11, p < 0.001), (a.u., arbitrary units).

CD, quantified as MPI, was found to correlate positively with the percentage of spurious brightness originating from the iris (*r* = 0.47, *β* = 1.49, *p* < 0.001), as shown in Fig. 4a. This suggests that increased reflection from the iris leads to artificially elevated CD measurements. The color gradient in Fig. 4a further highlights the role of iris pigmentation: lighter irises (lower *IrisColor* values) tend to present both more reflection and higher CD values.

**Fig. 5:**
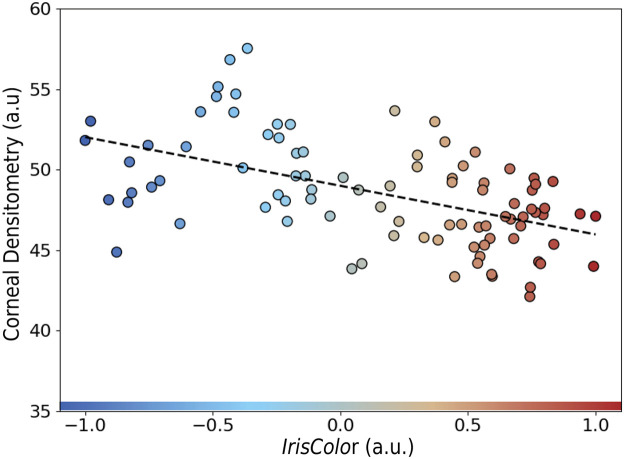
Correlation between corneal densitometry and IrisColor (r = –0.52, β = –1.08, p < 0.001), (a.u., arbitrary units).

This effect is directly linked to iris pigmentation, as demonstrated by a strong negative correlation between *IrisColor* and iris spurious brightness (*r* = –0.83, *β* = –1.11, *p* < 0.001), illustrated in Fig. 4b. Lighter-colored irises consistently exhibited higher levels of reflected brightness. In line with this, a significant negative correlation was also observed between *IrisColor* and CD (*r* = –0.52, *β* = –1.08, *p* < 0.001) as shown in Fig. 5, confirming that iris pigmentation has a measurable influence on CD outcomes.

These findings are further supported by the groupwise analysis presented in Table 1. Eyes with light-colored irises (defined by negative *IrisColor* values) showed significantly higher CD, greater percentages of iris-related brightness artefacts, and increased total image brightness.

**Table 1:** Comparison of corneal densitometry (CD), computed as mean pixel intensity (MPI), and brightness artefacts between eyes with light- and dark-colored irises. Values represent the mean ± SD of CD and the percentage of brightness artefacts originating from the iris, lateral regions, and the overall Scheimpflug image. p-values were derived from linear mixed-effects models (LMMs).

	CD cornea (a.u.)	% Iris brighness	% Lateral brighness	% Overall brighness
Light-colored irises (n=39)	50.3 ± 3.2	2.3 ± 0.7	3.9 ± 0.7	6.5 ± 0.9
Dark-colored irises (n=52)	47.2 ± 2.6	0.8 ± 0.5	3.4 ± 0.7	4.3 ± 0.8
p-value (LMMs)	< 0.001	<0.001	0.04	<0.001

**Fig. 6:**
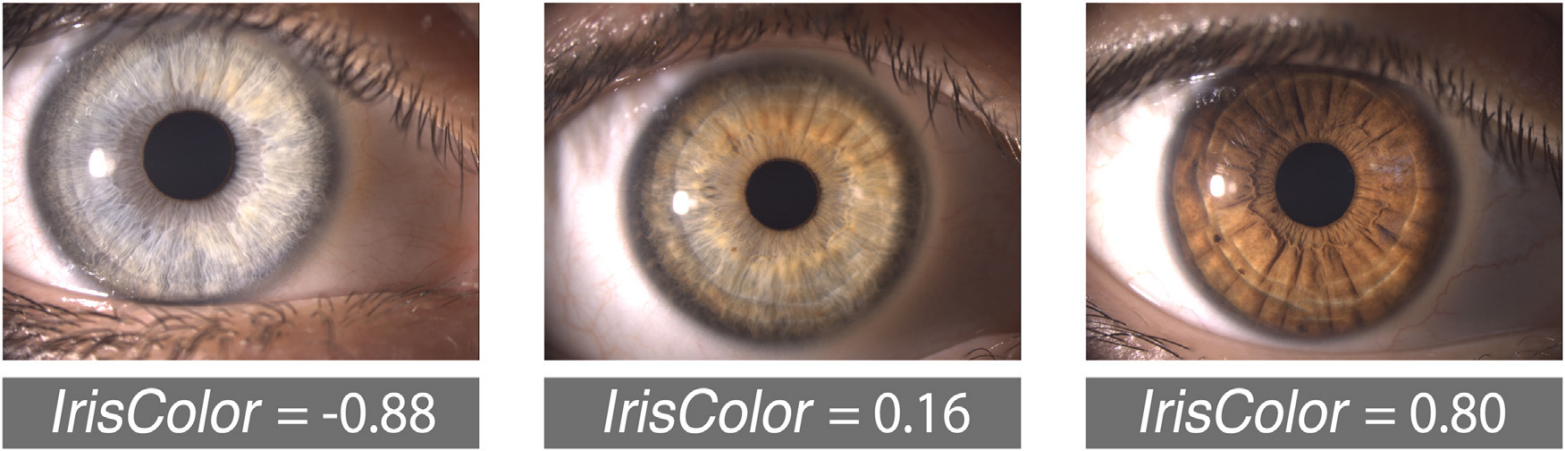
Representative examples of eyes with light, intermediate, and dark iris pigmentation, illustrating differences in the IrisColor coefficient. The numbers indicate the corresponding IrisColor values, with lower (negative) values representing lighter irises and higher (positive) values representing darker irises.

Fig. 6 illustrates three representative slit-lamp images corresponding to different *IrisColor* values. This visual example highlights how the proposed metric captures the continuous variation in iris pigmentation, from light (negative values) to dark (positive values).

## Discussion

This study demonstrates that iris pigmentation introduces a measurable bias of approximately 3.1 units in CD values obtained via Scheimpflug imaging, equivalent to a 6.6% relative overestimation in eyes with light-colored irises (Table 1). CD was positively correlated with the amount of reflected brightness from the iris (*r* = 0.47, *β* = 1.49, p < 0.001, Fig. 4a), which in turn was strongly dependent on iris pigmentation (*r* = –0.83, *β* = 1.49, p < 0.001, Fig. 4b). Eyes with lighter irises exhibited significantly higher CD values, driven primarily by increased brightness artefacts originating from the iris. To our knowledge, this is the first study to formally quantify how iris pigmentation affects CD.

By introducing a continuous and objective metric (*IrisColor*) based on the CIELAB color space, we were able to characterize iris pigmentation without relying on subjective classification. While previous studies have also used CIELAB components to quantify iris color, they typically analyzed each channel (L*, a*, b*) independently.^18^^,^^19^ This fragmented approach limits the ability to capture overall iris pigmentation and hampers its clinical applicability, as it fails to provide a unified representation of eye color. In contrast, the proposed *IrisColor* metric combines normalized luminance and chromaticity information into a single scalar value. It also captures variations in image illumination, an important advantage in clinical environments, where lighting conditions are often suboptimal or difficult to control despite standardization efforts. Negative values of the *IrisColor* metric indicate light-colored irises, positive values correspond to dark-colored irises, and values near zero typically reflect ambiguous or mixed pigmentation, which are often difficult to categorize subjectively. This is exemplified in the central image of Fig. 6, where the iris displays both bluish and brownish hues, illustrating the added value of an objective, continuous classification over traditional categorical approaches.

Previous studies have identified age as a factor influencing CD values.^7^^,^^8^ For this reason, participants in the current study were selected within a limited age range and were uniformly distributed across iris color groups, thereby minimizing the potential confounding effect of age. Moreover, all subjects were healthy young adults with no history of ocular pathology, and slit-lamp examination confirmed the absence of any corneal opacities or abnormalities. It is therefore important to emphasize that the observed increase in CD in eyes with lighter irises does not reflect a true reduction in corneal transparency, but rather an artefactual elevation caused by secondary light reflected from the iris and captured within the Scheimpflug image. This effect is inherent to Scheimpflug-based imaging systems, which rely on slit-beam illumination projected obliquely across the anterior segment. Light reflected from highly scattering or bright structures, such as lightly pigmented irises, can reach the camera sensor indirectly, producing diffuse illumination that overlaps with the corneal region in the image. When applying a fixed image-processing pipeline, this unintended light contribution increases the pixel intensity within the segmented corneal area, thus leading to an overestimation of CD.

In our previous work, we found that lateral brightness artefacts originating from peripheral regions such as the anterior sclera had a stronger influence on CD than iris-related artefacts, with r = 0.42 (p < 0.05) compared to r = 0.34 (p < 0.05) for iris brightness.^10^ Lateral artefacts also represented a larger portion of the total spurious brightness detected in Scheimpflug images (4% vs. 2% on average). However, the current findings suggest that even these peripheral artefacts are not entirely independent of iris pigmentation: eyes with lighter irises showed slightly higher lateral brightness values (p = 0.04, Table 1). While this association is statistically weak, it raises the possibility that overall image brightness, including reflections from anatomical regions beyond the iris, may be modulated by pigmentation-related optical properties, such as back-scattered light within the anterior segment.

This study does not present major limitations. Not all participants contributed both eyes, and although mixed-effects models were used to account for intra-subject correlation, some residual inter-eye variability may persist. Additionally, the sample consisted of healthy young adults within a narrow age range, which may limit the generalizability of the findings to older populations or eyes with ocular pathology. Moreover, all images were acquired using a single Scheimpflug system under standardized conditions. While this improves internal consistency, it may limit generalizability to other devices or imaging protocols.

The findings of the current work have important implications for the clinical interpretation of CD, which is increasingly used as a diagnostic biomarker in ophthalmology. CD has been applied to detect early signs of keratoconus,^5^^,^^6^ monitor postoperative healing,^3^^,^^4^ and evaluate subtle alterations in corneal transparency following refractive surgery.^1^^,^^2^ In this study, all eyes analyzed belonged to young, healthy individuals with no history of ocular pathology or surgery, and therefore represent an optimal standard of corneal transparency. Despite this, Fig. 4a shows differences in CD exceeding 10% between eyes with different iris pigmentation, solely due to brightness artefacts. Such differences, if not properly accounted for, could falsely suggest the presence of corneal opacification or progression of disease in clinical or research settings that rely on CD thresholds. This issue is further compounded by the uneven global distribution of iris pigmentation. Light-colored irises are more prevalent in Northern European populations, while dark irises dominate in Asia, Africa, and Latin America. As such, CD-based diagnostic criteria developed in one population may not generalize to others, potentially introducing bias in multiethnic studies or in clinical practice across different regions of the world.

In conclusion, our findings indicate that iris pigmentation is a relevant and clinically overlooked source of variability in Scheimpflug-based CD. As CD continues to gain traction as a diagnostic and monitoring tool, future work should explore whether normalization strategies, device-specific corrections, or pigmentation-adjusted models can reduce this bias and enhance the clinical utility of CD metrics.

## Declaration of competing interest

Authors report no conflict of interest.
